# Factors associated with adherence to allocated treatment in the ASCEND trial: a mail-based randomised trial of aspirin and of omega-3 fatty acid supplementation in people with diabetes

**DOI:** 10.1186/s13063-026-09551-4

**Published:** 2026-02-27

**Authors:** Vichithranie W. Madurasinghe, Marion Mafham, Georgina Buck, Louise Bowman, Jane Armitage

**Affiliations:** Clinical Trial Service Unit and Epidemiological Studies Unit, Oxford Population Health, Richard Doll Building, Old Road Campus, Oxford, OX3 7LF UK

**Keywords:** Aspirin, Omega-3 fatty acids, Diabetes, Cardiovascular disease, Randomised controlled trial, Adherence

## Abstract

**Introduction:**

ASCEND assessed the effects of randomisation to aspirin versus placebo and, separately omega-3 fatty acid (FA) supplementation versus placebo on vascular events in 15480 adults with diabetes using mail-based remote methods. This analysis investigates factors associated with adherence to the allocated treatments.

**Methods:**

Adherence was estimated from the 6-monthly follow-up forms and the supply of study treatment packs. A binary adherence variable, full versus less than full adherence during the period at risk of a serious vascular event (SVE), was investigated using logistic regression. Potential predictors of adherence considered were sex, age at randomisation, Hospital Frailty Risk Score, ethnicity, Townsend index, smoking status, type of diabetes, predicted 5-year vascular risk, number of other medications reported at study entry and treatment allocation.

**Results:**

Seven thousand, three hundred twelve (47.2%) participants were fully adherent to aspirin/placebo and 8937 (57.7%) were fully adherent to omega-3 FA/placebo while at risk of a SVE. Women were less likely to be fully adherent than men (aspirin randomisation: 2509 [43.3%] vs. 4803 [49.6%]; OR, 0.73; 95% CI: 0.68 to 0.80; *P* < 0.0001; omega-3 FA randomisation: 3035 [52.4%] vs. 5902 [61.0%]; OR, 0.67; 95% CI: 0.61 to 0.72; *P* < 0.0001) and adherence decreased with increasing frailty (trend *p*-value < 0.0001), smoking (*p*-value < 0.0001), vascular risk score (*p*-value < 0.0001) and deprivation (trend *p*-value = 0.0211, *p* = 0.0011).

**Conclusion:**

We show this large, mail-based trial has comparable adherence with less streamlined trials in similar populations. In ASCEND, women and those with higher levels of frailty, smoking and vascular risk score had poorer adherence to trial medication. Trialists should consider strategies to improve adherence in these participant groups.

**Supplementary Information:**

The online version contains supplementary material available at 10.1186/s13063-026-09551-4.

## Introduction

ASCEND assessed the efficacy and safety of aspirin and omega-3 fatty acid [FA] supplementation for primary prevention of cardiovascular events in 15480 men and women, age 40 years or older with a diagnosis of diabetes mellitus (any type) and without known cardiovascular disease (CVD) in a randomised 2 × 2 factorial blinded design [1, 2]. In order to be cost-effective, ASCEND participant recruitment and follow up procedures were highly streamlined and were undertaken predominantly by mail [3]. Potentially eligible participants were identified from centrally-held registers and general practitioner held disease registers and invited to take part. Those willing and eligible to participate entered a pre-randomisation run-in phase and were mailed a pack of medication containing placebo tablets and capsules. Participants who remained eligible and willing to continue at the end of the run-in period were randomised. After randomisation, participants were mailed study medication and follow-up questionnaires every 6 months until the end of the trial. The average follow up was 7.4 years and at the end of the scheduled follow-up period, complete follow-up data were available for 15,341 (99.1%) of those randomised. The primary outcome was serious vascular events (SVE) defined as a composite of non-fatal myocardial infarction, non-fatal stroke excluding confirmed intracranial haemorrhage, transient ischemic attack or death from any vascular cause excluding confirmed intracranial haemorrhage. During the study several strategies were employed to encourage adherence with study treatment including questionnaire reminders, telephone interaction with coordinating centre staff, regular newsletters and the use of the pre-randomisation run-in. Of those who entered the single-blind placebo run-in period in ASCEND, approximately 40% dropped out of the study before randomisation. Had there been no run-in, these withdrawals would probably have occurred after randomisation (most likely in the first 6–12 months), thereby substantially reducing the statistical power of the study [3].

In ASCEND the estimated mean adherence (weighted according to person-years at risk of a SVE) was 70% in the active and placebo aspirin arms, and 77% among those allocated omega-3 FA arm vs 76% allocated placebo capsules; and was found to decline over time from randomisation (from > 85% in the first 3 years to around 60% at ≥ 7 years), with the main reason for stopping being participant choice [2, 4]. The main results showed that aspirin reduced SVEs (658 [8.5%] and 743 [9.6%] aspirin vs placebo; rate ratio [RR], 0.88; 95% confidence interval [CI], 0.79 to 0.97; *P* = 0.01), but aspirin also caused major bleeding events (314 [4.1%] vs 245 [3.2%]; RR 1.29; 95% CI, 1.09 to 1.52; *P* = 0.003) [2]. There were no significant benefits or harms from omega-3 FA supplementation [4].

Poor adherence to allocated treatment is an important contributory factor in the failure of trials to obtain clear results. Lack of adherence tends to lead to underestimation of treatment effects, both in terms of events avoided and adverse events caused. In addition, systematic differences across trial arms in terms of numbers and characteristics of those who stop taking study treatment can lead to biased findings if this affects completeness of follow-up or leads to under-reporting of events. While mail-based trials like ASCEND can reduce the cost of running trials and the burden of taking part in trials, concerns about non-adherence to study treatments may limit the wider adoption of such streamlined methods. Identifying the baseline factors associated with poor adherence offers the opportunity to target adherence improvement strategies to the participant groups who could benefit the most. The aim of this analysis is to investigate such factors in the ASCEND trial.

## Methods

Adherence to allocated study treatment was assessed from 6-monthly follow-up questionnaires and from records of the supply of study treatment packs. Percentage of days a participant reported taking treatment out of the total days they were at risk of a SVE during the study was calculated for each participant and was found to have a highly negatively skewed distribution, with the largest group being adherent on 100% of days at risk (see supplementary figure S1 and S2). Therefore, adherence was analysed as a binary variable comparing adherence on 100% of days at risk (“full adherence”) versus adherence on less than 100% of days. A sensitivity analysis using a cut-off of adherence on 70% or more versus less than 70% of days at risk was also carried out.

Baseline participant characteristics and number of days at risk of a SVE were supplemented with linked healthcare data on hospitalisations to derive a Hospital Frailty Risk Score; this used hospitalisation occurring within the two years preceding randomisation [5]. Factors associated with adherence to aspirin vs placebo and, separately, to omega-3 FA supplementation vs placebo were assessed using a logistic regression model including the following covariates: sex, age at randomisation (< 60; ≥ 60 < 70; ≥ 70 years), Hospital Frailty Risk Score (0, > 0 ≤ 5, > 5), ethnicity, Townsend index at recruitment (a measure of deprivation) [3], smoking status at recruitment, type of diabetes, baseline vascular risk score (predicted 5-year risk of SVE without aspirin or omega-3 FA < 5%, ≥ 5% to < 10%, or ≥ 10% [2]), number of other medications at study entry and treatment allocation. The covariates included in the model were selected a priori based on our experience in our previous cardiovascular trials. All randomised participants were included. All analyses were performed in SAS 9.4 (SAS Institute, Cary NC). Logistic regression used the GENMOD procedure to model full adherence as the outcome event with a logit link and estimated the overdispersion factor from the deviance divided by degrees of freedom. The option aggregate was specified to assess deviance and Pearson's Chi-square statistics. The resulting maximum likelihood parameter estimates are reported as odds ratios (OR) and 95% confidence intervals (CI). To avoid restricting comparisons to an arbitrary reference group, floating absolute risk CIs were calculated where a covariate has three or more categories [6]. Tests for heterogeneity were used to determine whether the effects in subgroups differed from the overall effect. When such subgroups could be arranged in some meaningful order (e.g. age at randomisation), assessment of any trend in the proportional effects on outcome was reported. Analysis focused on main effects only, however additional models tested whether any first order interactions were statistically significant. The statistical significance of main effects was assessed at the 5% level (two-sided) using p-values from type 3 analysis (i.e. after allowance for all other effects in the model). We made allowance for multiple hypothesis testing in the interpretation of the results but made no formal adjustments to the p values. First order interactions were only reported if they were statistically significant at 2p < 0.001. The model goodness-of-fit was assessed using Pearson chi-square statistic.

## Results

Of the 15,480 participants randomised, around one third of study participants (37.4%) were women and nearly a quarter (23.5%) were aged 70 years or older. The majority of participants were white (96.5%) and had a Townsend deprivation score below two (85.3%) indicating they were living in less deprived areas. Only 11.4% of participants had a frailty score above zero indicating some level of frailty and 17.2% had a high predicted vascular risk of ≥ 10% at 5 years. Participants were taking an average of 5.5 other medications when they entered the trial.

Although study average adherence was 70% in the aspirin and placebo arms [2] only 47.2% were fully adherent to aspirin or placebo (i.e. adherent on all days they were at risk of SVEs) (Table 1). Similarly, study average adherence was 77% and 76% to omega-3 FA and placebo capsules respectively [4] but only 57.7% were fully adherent on all days at risk of SVEs (Table 1). The Pearson chi-square/degrees of freedom for the models of main effects were 1.03 for aspirin/placebo and 0.99 for omega-3 FA/placebo indicating that goodness-of-fit was good. Full adherence to study medication was less likely in women than men (aspirin/placebo 43.3% vs. 49.6%, OR 0.73, 95% CI 0.68–0.80; omega-3 FA/placebo 52.4% vs. 61.0%; OR 0.67, 95% CI 0.61–0.72) (Table 1). Compared with never smokers, current (aspirin/placebo 39.7% vs. 49.5%, OR 0.69, 95% CI 0.61–0.80; omega-3 FA/placebo 48.6% vs. 59.3%; OR 0.67, 95% CI 0.59–0.77) and former (aspirin/placebo 46.4% vs. 49.5%, OR 0.87, 95% CI 0.82–0.92; omega-3 FA/placebo 57.8% vs. 59.3%; OR 0.89, 95% CI 0.84–0.94) smokers were slightly less adherent to study treatments (Table 1). Furthermore, adherence decreased with increasing frailty (trend *p*-value < 0.0001, *p* < 0.0001) and baseline vascular risk score (trend *p*-value < 0.0001, *p* = 0.0001). There was some evidence that higher levels of deprivation (trend *p*-value = 0.0211, *p* = 0.0011) may also be associated with lower adherence. There was inconsistent evidence of an association with age (trend *p*-value = 0.0270, *p* = 0.5258), ethnicity (*p* = 0.1552, *p* = 0.0185) and number of other medications at study entry (trend *p*-value = 0.0007, *p* = 0.5637). Type of diabetes and treatment allocation were not clearly associated with adherence. The sensitivity analysis (comparing adherence on ≥ 70% versus < 70% of days) gave similar results to the main results (see supplementary material, Table S1) and none of the first order interactions were statistically significant at 2p < 0.001.

**Table 1:** Adherence to study treatment

	Number of participants	Person years of follow up whilst at risk of a SVE—mean (standard deviation)	Aspirin comparison			Omega-3 fatty acid comparison		
			Fully adherent – number (%)	Odds ratio (floating absolute risk 95% Confidence Interval^*^)	Heterogeneity or trend *p*-value	Fully adherent – number (%)	Odds ratio (floating absolute risk 95% Confidence Interval^+^)	Heterogeneity or trend p-value
Overall	15480	7.14 (1.98)	7312 (47.2)			8937 (57.7)		
Demographic characteristics								
Sex								
Male	9684	7.04 (2.01)	4803 (49.6)	1.00 (1.00—1.00)	< .0001	5902 (61.0)	1.00 (1.00 – 1.00)	< .0001
Female	5796	7.30 (1.93)	2509 (43.3)	0.73 (0.68—0.80)		3035 (52.4)	0.67 (0.61—0.72)	
Age (years)								
Age < 60	5590	7.67 (1.88)	2813 (50.3)	1.00 (0.91—1.09)	0.0270^†^	3275 (58.6)	1.00 (0.91—1.09)	0.5258^†^
Age > = 60, < 70	6247	7.03 (1.86)	3088 (49.4)	1.05 (1.01—1.09)		3812 (61.0)	1.17 (1.13—1.21)	
Age > = 70	3643	6.49 (2.13)	1411 (38.7)	0.79 (0.70—0.89)		1850 (50.8)	0.87 (0.77—0.98)	
Ethnicity								
White	14,935	7.13 (1.99)	7077 (47.4)	1.00 (0.95—1.05)	0.1552	8653 (57.9)	1.00 (0.95—1.05)	0.0185
African/Caribbean	140	7.05 (1.96)	49 (35.0)	0.66 (0.44—0.98)		57 (40.7)	0.55 (0.38—0.82)	
Indian/Pakistani/Bangladeshi	184	7.43 (1.52)	85 (46.2)	0.86 (0.61—1.20)		108 (58.7)	0.95 (0.68—1.32)	
Other/unknown	221	7.22 (1.63)	101 (45.7)	0.91 (0.67—1.24)		119 (53.9)	0.84 (0.62—1.14)	
Townsend Index								
TI < -3	5104	7.11 (1.98)	2463 (48.3)	1.00 (0.94—1.07)	0.0211^†^	3043 (59.6)	1.00 (0.94—1.07)	0.0011^†^
TI > = -3, < 0	6060	7.15 (1.94)	2915 (48.1)	1.01 (0.95—1.07)		3527 (58.2)	0.96 (0.91—1.02)	
TI > = 0, < 2	2037	7.15 (2.01)	952 (46.7)	0.98 (0.89—1.09)		1159 (56.9)	0.94 (0.85—1.04)	
TI > = 2, < 4	1315	7.11 (2.08)	574 (43.7)	0.89 (0.78—1.01)		706 (53.7)	0.85 (0.75—0.96)	
TI > = 4, < 6	703	7.12 (2.08)	297 (42.3)	0.83 (0.70—0.99)		371 (52.8)	0.81 (0.68—0.96)	
TI > = 6	261	7.34 (2.26)	111 (42.5)	0.87 (0.65—1.16)		131 (50.2)	0.75 (0.57—1.00)	
Smoking status								
Never	6977	7.26 (1.91)	3454 (49.5)	1.00 (0.94—1.06)	< .0001	4140 (59.3)	1.00 (0.94—1.06)	< .0001
Former	7224	7.03 (2.00)	3350 (46.4)	0.87 (0.82—0.92)		4176 (57.8)	0.89 (0.84—0.94)	
Current	1279	7.05 (2.24)	508 (39.7)	0.69 (0.61—0.80)		621 (48.6)	0.67 (0.59—0.77)	
Clinical characteristics at baseline								
Type of diabetes								
Type 2	14,569	7.08 (1.96)	6844 (47.0)	1.00 (1.00—1.00)	0.2180	8385 (57.6)	1.00 (1.00 – 1.00)	0.1853
Type 1	911	8.02 (2.19)	468 (51.4)	1.11 (0.94—1.30)		552 (60.6)	1.12 (0.95—1.31)	
^+^Hospital Frailty Score								
Frailty score = 0	13,710	7.17 (1.96)	6615 (48.3)	1.00 (0.96—1.04)	< .0001^†^	8042 (58.7)	1.00 (0.96—1.04)	< .0001^†^
0 < Frailty score < = 5	1605	6.94 (2.16)	637 (39.7)	0.76 (0.68—0.85)		812 (50.6)	0.76 (0.68—0.86)	
Frailty score > 5	165	6.32 (2.06)	60 (36.4)	0.69 (0.48—1.00)		83 (50.3)	0.78 (0.55—1.10)	
Baseline vascular risk score								
Low (< 5%)	6264	7.58 (1.77)	3237 (51.7)	1.00 (0.91 – 1.10)	< .0001^†^	3781 (60.4)	1.00 (0.91—1.10)	0.0001^†^
Moderate (> = 5%, < 10%)	6548	7.02 (1.96)	3057 (46.7)	0.84 (0.83—0.85)		3799 (58.0)	0.87 (0.86—0.88)	
High (> = 10%)	2668	6.36 (2.23)	1018 (38.2)	0.69 (0.61—0.79)		1357 (50.9)	0.73 (0.65—0.83)	
Treatment and concomitant medication characteristics								
Allocated treatment								
Placebo	7740	7.11 (2.03)	3624 (46.8)	1.00 (1.00—1.00)	0.3990	4452 (57.5)	1.00 (1.00 – 1.00)	0.6766
Aspirin/Omega-3	7740	7.16 (1.94)	3688 (47.7)	1.03 (0.96 – 1.11)		4485 (58.0)	1.02 (0.94—1.09)	
Number of other medications at trial entry *(self-reported)*								
Zero	121	7.43 (1.80)	57 (47.1)	0.89 (0.59—1.34)	0.0007^†^	65 (53.7)	0.85 (0.57—1.28)	0.5637^†^
1	484	7.21 (1.85)	238 (49.2)	1.00 (0.81—1.23)		274 (56.6)	1.00 (0.81—1.23)	
2 to 3	3203	7.28 (1.87)	1604 (50.1)	1.05 (0.97—1.14)		1888 (58.9)	1.12 (1.03—1.22)	
4 to 5	4855	7.22 (1.95)	2366 (48.7)	1.05 (0.98—1.12)		2834 (58.4)	1.14 (1.07—1.22)	
6 to 7	3635	7.01 (2.02)	1729 (47.6)	1.05 (0.98—1.14)		2161 (59.5)	1.26 (1.17—1.36)	
8 to 9	1925	7.03 (2.04)	836 (43.4)	0.92 (0.82—1.02)		1070 (55.6)	1.11 (1.00—1.23)	
10 or more	1257	6.90 (2.21)	482 (38.4)	0.79 (0.69—0.90)		645 (51.3)	1.00 (0.88—1.14)	

^*^presented when there are more than two categories

^+^Hospital Frailty Score was calculated from linked Hospital Episode Statistics (or similar data in Scotland and Wales) following the methods described in Lancet 2018; 391: 775–82

^†^trend test

## Discussion

Using a factorial design and streamlined procedures, ASCEND produced reliable evidence for the effects of aspirin for primary cardiovascular prevention in adults with diabetes [2, 4]. The estimated *average* adherence to aspirin or placebo during the study was 70%, and 77% to the omega 3 FA capsules or 76% to placebo. However, in this analysis we show that only 47% and 58% respectively were fully adherent (i.e. adherent with their allocated tablets or capsules on all days spent at risk of a SVE during follow up). We show that sex is a major indicator of poorer adherence with women being one quarter to one third less likely to be adherent to treatment than men (OR 0.73 for aspirin adherence, OR 0.67 for omega-3 FA adherence). We also show that other factors including frailty, smoking and deprivation are associated with poorer adherence.

Previous studies examining factors associated with adherence to allocated treatment in CVD prevention trials have also indicated that women are less likely to adhere to study medication [7, 8]. Lau et al. [7] studied factors associated with adherence in 187,691 patients (28% women) enrolled in eleven phase 3 and 4 CVD trials conducted by the Thrombolysis in Myocardial Infarction (TIMI) group. Across these studies 26% of men (range 11%–37%) and 30% of women (range 16%–49%) discontinued the study drug prematurely. Patient-level meta-analysis results showed that women had 27% higher overall odds of permanent study drug discontinuation versus men (odds ratio [OR], 1.27, 95% CI 1.17–1.36, *p* < 0.001) and female sex was still associated with treatment discontinuation after accounting for baseline differences (OR, 1.22, 95% CI 1.16–1.28, *p* < 0.001). Our finding of a similar impact of sex on adherence in a mail-based study suggests that site-related factors (including reluctance to attend face-to-face visits) are not major determinants of lower adherence among women.

Other factors shown to be associated with poor adherence to allocated treatment in CVD prevention trials include age, race, body mass index, cigarette smoking, lack of exercise, family history of myocardial infarction, history of angina, hypertension, and high cholesterol [9–11].

Brunner et al. [11] found an association between ethnicity and treatment adherence in the Women’s Health Initiative (WHI) trial of calcium and vitamin D (CaD) but despite some suggestion that ethnicity may be associated with poorer adherence, ASCEND included too few participants of different ethnicities to reliably assess this. The ASCEND data showed that frailty and deprivation were also associated with poorer adherence. It is well recognised that socioeconomic circumstances are associated with health outcomes in the general population with poorer individuals experiencing worse health compared to their more well off counterparts [12]. Some studies suggest that socioeconomic factors are also associated with adherence to prescribed medication in routine clinical practice [13]. In the context of clinical trials, Rae et at. [14] found that inequalities in socioeconomic and geographic factors influence referral and enrolment to early phase clinical trials of treatments for cancer. However currently there is little evidence that socioeconomic factors are associated with clinical outcomes or adherence to allocated treatment in participants enrolled in cardiovascular trials [13] although Lemstra et al. [15] found that primary prevention (rate ratio = 1.52; 95% CI, 1.50–1.53) and lower income status (rate ratio = 1.26; 95% CI, 1.16–1.37) were both independent predictors of non-adherence to statin medications in clinical practice.

In ASCEND, increased frailty was associated with poorer adherence to allocated treatment despite individuals with a higher frailty score having fewer person years at risk of an SVE. To our knowledge, the role of frailty on adherence in CVD primary prevention trials has not been previously assessed. However, it is important to recognise that, while the Hospital Frailty Score [5] has been validated in hospitalised adults of any age [16], it was not designed for use in non-hospitalised populations. However, linked national data on hospital admissions was available for ASCEND participants in England, Wales and Scotland for 5 years prior to their trial participation, allowing calculation of the score using International Statistical Classification of Diseases and Related Health Problems (ICD-10) codes and similar national data in Denmark has shown that the score is associated with co-morbidities, complications and medication use among people with diabetes or cardiovascular disease [17].

We only considered a limited number of factors in this analysis, which were felt likely to be relevant for identifying groups of participants with poorer treatment adherence in a mail-based trial. It is likely that there are non-clinical and less tangible factors (e.g. participant fatigue, loss of motivation) associated with decline in treatment adherence over time, however our aim was to identify participant groups *at baseline* that may benefit from strategies to improve adherence. Studies evaluating adherence to preventative medications prescribed in routine clinical practice have also found that adherence to medications is poor in cardiovascular patient populations, particularly among those who have not had a previous myocardial infarction. Naderi and colleagues [18] reported that just over half (57%; 95% CI, 50% to 64%) were adherent (patients possessing medication more than approximately 75% of the time were judged to be adherent) after a median of 24 months. There were statistically significant differences between primary and secondary prevention populations with only 50% (CI, 45%-56%) and 66% (CI, 56%-75%) of patients taking medications as prescribed long-term, (*p* = 0.012). A population-based cohort study [19] examining the long-term (up to 10 years) adherence to low dose aspirin for primary/secondary prevention of CVD found that the median adherence was 50% in Germany and United Kingdom.

Average adherence in ASCEND (70% in aspirin and placebo arms) was comparable with other trials assessing aspirin for primary prevention of CVD [10, 11, 20–26]. A meta-analysis of trials of aspirin in CVD primary prevention showed that aspirin use was associated with significant reductions in the composite outcome of cardiovascular mortality, myocardial infarction and stroke [27, 28], but adherence with aspirin therapy in the intervention group ranged from 50 to 90 percent with most reporting declining adherence with longer duration of follow up [27]. But these studies did not report on factors associated with less good adherence.

ASCEND was a large UK trial, with relatively small numbers of very old, frail or participants of non-white ethnicity. Hence our findings may not be applicable to trials enrolling more diverse populations or to other country settings. In ASCEND, several strategies were employed post-randomisation to encourage adherence with study treatment, including questionnaire reminders, telephone interaction with coordinating centre staff and regular newsletters. However, we have not attempted to assess these methods formally and are unable to comment on which had the most impact.

This study has a number of limitations. First, we were unable to examine factors related to early vs late discontinuation of study treatment. Since participants may stop and restart study treatment during follow-up during a long-term study, we chose to examine the overall percentage of days at risk of a serious adverse event that the participant was adherent to study medication. This provided the most accurate estimate of overall adherence but meant that a time to discontinuation analysis was not possible. Second, information on adherence was obtained from participant self-report on 6-monthly follow-up questionnaires, along with treatment supply records, which could introduce recall and desirability bias. However, alternative methods, such as pill counting, were not feasible in a highly streamlined mail-based phase 4 study. However, a sub-study of ASCEND was conducted in 152 randomly selected participants (76 aspirin vs 76 placebo) where urinary excretion of 11-dehydro-thromboxane B2 (UTxB2), a biomarker of adherence to aspirin, was assayed at baseline and a mean of two years after randomisation. The results showed 82% allocated aspirin achieved effective suppression indicating that they were taking aspirin as per trial allocation [29]. These findings are in line with adherence data from participant self-reports. Third, the use of the pre-randomisation placebo run-in period was used to identify individuals who were likely to stop study treatment after randomisation, resulting in a population selected for better adherence. This may limit direct application of the finding of this study to trials without a run-in period. However, the benefits of a pre-randomisation run-in ensuring high levels of adherence to study treatment in the randomised population and consequent improvements in statistical power are well established [30]. Finally, the ASCEND trial population, recruited between 2005 and 2011, was predominantly drawn from individuals of White British ethnicity and, therefore, this study is unable to assess the effect of ethnicity on adherence or assess factors that might be of particular relevance to ethnic minority groups.

## Conclusion

We show that ASCEND has comparable adherence with less streamlined trials in similar populations indicating that wider adoption of ASCEND streamlined methods can reduce the running cost and the burden of taking part in trials without a detrimental effect on adherence. Our findings provide new insights into factors associated with adherence, with females, smokers and those with a high frailty and vascular risk more likely to have poorer adherence to trial medication. Future trials should consider tailoring their treatment adherence strategies to address the needs of specific participant groups in an effort to improve adherence.

## Supplementary Information

Supplementary Material 1.

## Data Availability

Proposals for data access will be considered by the ASCEND Steering Committee in accordance with the trial protocol. Procedures for accessing the data are available at: https://www.ndph.ox.ac.uk/data-access#:~:text=We%20welcome%20requests%20from%20researchers,these%20data%20for%20public%20health.

## Abbreviations

ASCEND: A Study of Cardiovascular Events iN Diabetes
CI: Confidence interval
CTSU: Clinical Trial Service Unit
CVD: Cardiovascular disease
FA: Fatty acid
MREC: Multi-centre Research Ethics Committee
MHRA: Medicines and Healthcare Products Regulatory Agency
OR: Odds ratio
PIAG: Patient Information Advisory Group
REC: Research Ethics Committee
RR: Rate ratio
SVE: Serious Vascular Event
