# Supplementary material for: Factors associated with adherence to allocated treatment in the ASCEND trial: a mail-based randomised trial of aspirin and of omega-3 fatty acid supplementation in people with diabetes
Source: Trials. 2026 Feb 27;27:183. doi: 10.1186/s13063-026-09551-4 (PMC12947497; doi:10.1186/s13063-026-09551-4)
Supplement: Supplementary file 1 — Supplementary Material 1. [file 13063_2026_9551_MOESM1_ESM.docx]

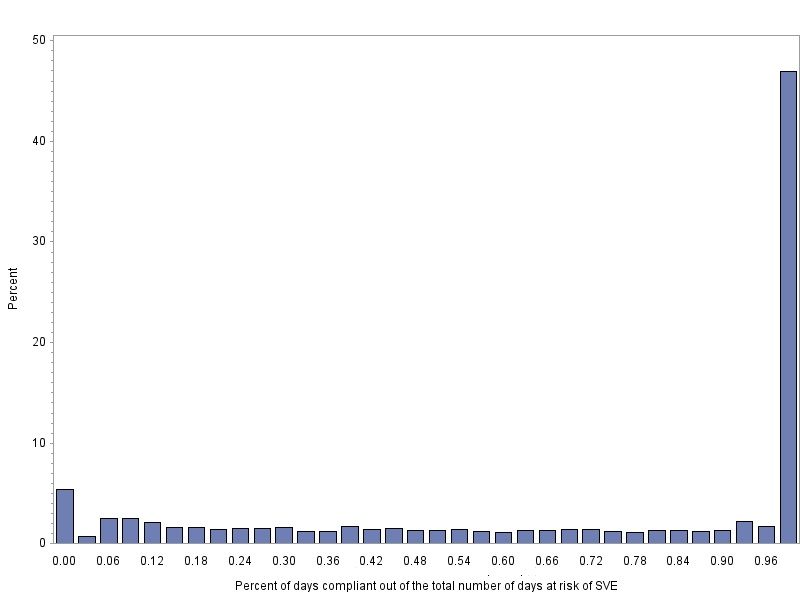
**Supplementary material**

Figure S1: Distribution of individual compliance, aspirin vs placebo


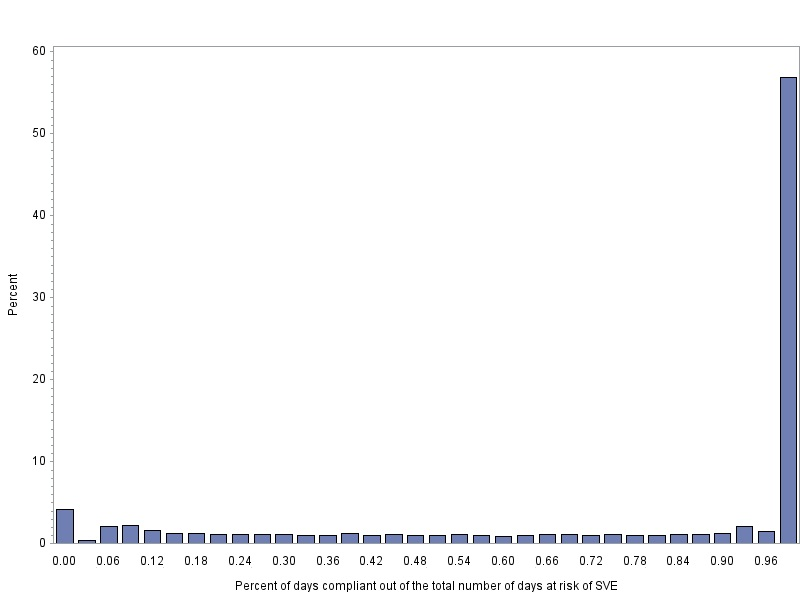


Figure S2: Distribution of individual compliance, omega-3 FA vs placebo

Table S1: Adherence to study treatment, adherent on at least 70% of days at risk of a SVE

|  | **Number of participants** | **Person years of follow up whilst at risk of a SVE - mean (standard deviation)** | **Aspirin comparison** | | | **Omega-3 fatty acid comparison** | | |
| --- | --- | --- | --- | --- | --- | --- | --- | --- |
|  |  |  | **70% adherent – number (%)** | **Odds ratio (floating absolute risk 95% Confidence Interval*)** | **Heterogeneity or trend p-value** | **70% adherent – number (%)** | **Odds ratio (floating absolute risk 95% Confidence Interval*)** | **Heterogeneity or trend p-value** |
| **Overall** | **15480** | **7.14 (1.98)** | **9471 (61.2)** |  |  | **10814 (69.9)** |  |  |
| **Sex** |  |  |  |  |  |  |  |  |
| Male | 9684 | 7.04 (2.01) | 6162 (63.6) | 1.00  (1.00 – 1.00) | <.0001 | 7054 (72.8) | 1.00  (1.00 – 1.00) | <.0001 |
| Female | 5796 | 7.30 (1.93) | 3309 (57.1) | 0.73  (0.67 - 0.79) |  | 3760 (64.9) | 0.65  (0.60 - 0.71) |  |
| **Age (years)** |  |  |  |  |  |  |  |  |
| Age <60 | 5590 | 7.67 (1.88) | 3485 (62.3) | 1.00  (0.91 - 1.10) | 0.3957^†^ | 3867 (69.2) | 1.00  (0.91 - 1.10) | 0.2686^†^ |
| Age >=60, <70 | 6247 | 7.03 (1.86) | 3963 (63.4) | 1.12  (1.08 - 1.16) |  | 4531 (72.5) | 1.23  (1.18 - 1.28) |  |
| Age >=70 | 3643 | 6.49 (2.13) | 2023 (55.5) | 0.87  (0.77 - 0.98) |  | 2416 (66.3) | 1.00  (0.89 - 1.13) |  |
| **Ethnicity** |  |  |  |  |  |  |  |  |
| White | 14935 | 7.13 (1.99) | 9157 (61.3) | 1.00  (0.95 - 1.05) | 0.1502 | 10456 (70.0) | 1.00  (0.95 - 1.05) | 0.1018 |
| African/Caribbean | 140 | 7.05 (1.96) | 67 (47.9) | 0.64  (0.44 - 0.95) |  | 78 (55.7) | 0.62  (0.42 - 0.90) |  |
| Indian/Pakistani/  Bangladeshi | 184 | 7.43 (1.52) | 111 (60.3) | 0.89  (0.63 - 1.25) |  | 131 (71.2) | 1.00  (0.70 - 1.43) |  |
| Other/unknown | 221 | 7.22 (1.63) | 136 (61.5) | 1.01  (0.73 - 1.38) |  | 149 (67.4) | 0.91  (0.66 - 1.25) |  |
| **Townsend Index** |  |  |  |  |  |  |  |  |
| TI <-3 | 5104 | 7.11 (1.98) | 3174 (62.2) | 1.00  (0.94 - 1.07) | 0.0029^†^ | 3657 (71.7) | 1.00  (0.93 - 1.07) | <.0001^†^ |
| TI >=-3, <0 | 6060 | 7.15 (1.94) | 3772 (62.2) | 1.02  (0.96 - 1.08) |  | 4279 (70.6) | 0.97  (0.91 - 1.03) |  |
| TI >=0, <2 | 2037 | 7.15 (2.01) | 1247 (61.2) | 1.00  (0.90 - 1.11) |  | 1410 (69.2) | 0.94  (0.84 - 1.04) |  |
| TI >=2, <4 | 1315 | 7.11 (2.08) | 748 (56.9) | 0.86  (0.75 - 0.97) |  | 858 (65.2) | 0.80  (0.71 - 0.91) |  |
| TI >=4, <6 | 703 | 7.12 (2.08) | 385 (54.8) | 0.78  (0.66 - 0.93) |  | 447 (63.6) | 0.74  (0.62 - 0.88) |  |
| TI >=6 | 261 | 7.34 (2.26) | 145 (55.6) | 0.83  (0.62 - 1.10) |  | 163 (62.5) | 0.73  (0.55 - 0.97) |  |
| **Smoking status** |  |  |  |  |  |  |  |  |
| Never | 6977 | 7.26 (1.91) | 4395 (63.0) | 1.00  (0.94 - 1.06) | <.0001 | 4953 (71.0) | 1.00  (0.94 - 1.06) | <.0001 |
| Former | 7224 | 7.03 (2.00) | 4381 (60.6) | 0.87  (0.83 - 0.92) |  | 5073 (70.2) | 0.90  (0.85 - 0.96) |  |
| Current | 1279 | 7.05 (2.24) | 695 (54.3) | 0.73  (0.64 - 0.83) |  | 788 (61.6) | 0.69  (0.61 - 0.80) |  |
| **Type of diabetes** | | | | | | | | |
| Type 2 | 14569 | 7.08 (1.96) | 8894 (61.0) | 1.00  (1.00 – 1.00) | 0.4586 | 10161 (69.7) | 1.00  (1.00 – 1.00) | 0.1493 |
| Type 1 | 911 | 8.02 (2.19) | 577 (63.3) | 1.07  (0.90 - 1.26) |  | 653 (71.7) | 1.14  (0.96 - 1.35) |  |
| **^+^Hospital Frailty Score** | | | | | | | | |
| Frailty score=0 | 13710 | 7.17 (1.96) | 8509 (62.1) | 1.00  (0.96 - 1.05) | <.0001^†^ | 9678 (70.6) | 1.00  (0.96 - 1.05) | 0.0001^†^ |
| 0<Frailty score<5 | 1605 | 6.94 (2.16) | 873 (54.4) | 0.77  (0.69 - 0.86) |  | 1031 (64.2) | 0.78  (0.70 - 0.88) |  |
| Frailty score>5 | 165 | 6.32 (2.06) | 89 (53.9) | 0.79  (0.55 - 1.13) |  | 105 (63.6) | 0.79  (0.55 - 1.13) |  |
| **Baseline vascular risk score** | | | | | | | | |
| Low (<5%) | 6264 | 7.58 (1.77) | 4008 (64.0) | 1.00  (0.90 - 1.11) | 0.0015^†^ | 4445 (71.0) | 1.00  (0.90 - 1.11) | 0.0025^†^ |
| Moderate (>=5%, <10%) | 6548 | 7.02 (1.96) | 3965 (60.6) | 0.85  (0.84 - 0.86) |  | 4590 (70.1) | 0.87  (0.86 - 0.88) |  |
| High (>=10%) | 2668 | 6.36 (2.23) | 1498 (56.1) | 0.79  (0.69 - 0.90) |  | 1779 (66.7) | 0.78  (0.68 - 0.89) |  |
| **Allocated treatment** | | | | | | | | |
| Placebo | 7740 | 7.11 (2.03) | 4709 (60.8) | 1.00  (1.00 – 1.00) | 0.4669 | 5375 (69.4) | 1.00  (1.00 – 1.00) | 0.3426 |
| Aspirin/Omega-3 | 7740 | 7.16 (1.94) | 4762 (61.5) | 1.03  (0.95 - 1.11) |  | 5439 (70.3) | 1.04  (0.96 - 1.12) |  |
| **Number of other medications at trial entry** *(self-reported)* | | | | | | | | |
| Zero | 121 | 7.43 (1.80) | 69 (57.0) | 0.82  (0.54 - 1.25) | 0.0847^†^ | 75 (62.0) | 0.70  (0.46 - 1.06) | 0.8861^†^ |
| 1 | 484 | 7.21 (1.85) | 294 (60.7) | 1.00  (0.81 - 1.24) |  | 334 (69.0) | 1.00  (0.80 - 1.24) |  |
| 2 to 3 | 3203 | 7.28 (1.87) | 2008 (62.7) | 1.10  (1.01 - 1.20) |  | 2233 (69.7) | 1.04  (0.96 - 1.14) |  |
| 4 to 5 | 4855 | 7.22 (1.95) | 3023 (62.3) | 1.13  (1.05 - 1.20) |  | 3429 (70.6) | 1.13  (1.05 - 1.21) |  |
| 6 to 7 | 3635 | 7.01 (2.02) | 2262 (62.2) | 1.17  (1.09 - 1.27) |  | 2610 (71.8) | 1.25  (1.15 - 1.36) |  |
| 8 to 9 | 1925 | 7.03 (2.04) | 1134 (58.9) | 1.05  (0.94 - 1.17) |  | 1314 (68.3) | 1.09  (0.98 - 1.22) |  |
| 10 or more | 1257 | 6.90 (2.21) | 681 (54.2) | 0.92  (0.81 - 1.05) |  | 819 (65.2) | 1.02  (0.90 - 1.17) |  |

*presented when there are more than two categories

^+^Hospital Frailty Score was calculated from linked Hospital Episode Statistics (or similar data in Scotland and Wales) following the methods described in Lancet 2018; 391: 775–82

^†^trend test
